# Guiding Documents for Disaster Risk Reduction and Management in Health Care System of Nepal

**DOI:** 10.31729/jnma.5041

**Published:** 2020-10-31

**Authors:** Suraj Rijal, Sunil Adhikari, Ashis Shrestha

**Affiliations:** 1 Department of General Practice and Emergency Medicine, Patan Academy of Health Sciences, Lagankhel, Lalitpur, Nepal

**Keywords:** *disaster*, *Nepal*, *preparedness*

## Abstract

The incidence of disaster events has increased over the years. Nepal is vulnerable to various kinds of natural disasters especially earthquakes and floods and infectious disease outbreaks like Dengue and Covid-19 pneumonia. So, it is important to review and know our existing disaster risk reduction and management plans, rules, and regulations of our country to improve disaster risk management for resilience and enhancing disaster preparedness for effective response and to "Build Back Better: in recovery rehabilitation and reconstruction." Nepal has sufficient guiding documents to guide disaster management.

## INTRODUCTION

The incidence of disaster events has increased over the years. In 2018, there were 315 natural disaster events recorded with 11,804 deaths, over 68 million people affected, and US$131.7 billion in economic losses across the world. The impact of disasters is highest in Asia and accounted for 45% of disaster events, 80% of deaths, and 76% of people affected. Globally 45% of deaths were due to earthquakes followed by 24% of deaths due to flooding. Flooding affected the highest number of people, accounting for 50% of the total affected, followed by storms which accounted for 28%.^1^

## BACKGROUND

So, a global consensus is required for the management of disaster, which is represented by the Sendai Framework (2015-2030). This framework applies to the risk of small-scale and large-scale, frequent and infrequent, sudden, and slow-onset disasters, caused by natural or manmade hazards as well as related environmental, technological and biological hazards and risks. It focuses on the implications of disaster on health and promotes health resilience.^2,3^ It aims to guide the multi-hazard management of disaster risk in development at all levels as well as within and across all sectors. Therefore it has listed four priority actions: understanding disaster risk, strengthening disaster risk governance to manage disaster risk, investing in disaster risk management for resilience and enhancing disaster preparedness for effective response, and to “Build Back Better: in recovery rehabilitation and reconstruction."

Disaster being a cross-cutting issue has also been addressed in sustainable development goals 2030. The SDG proposed for urgent action to combat climate changes and its impact (SDG 13), healthy lives (SDG 3), and making human settlements inclusive, safe, resilient, and sustainable (SDG11).^4^ Furthermore, 196 countries including all WHO Member States have agreed in the International Health Regulation (IHR) to work together for global health security. The purpose and scope of the IHR are to prevent, protect against, control and provide a public health response to the international spread of disease in ways that are commensurate with and restricted to public health risks, and which avoid unnecessary interference with international traffic and trade.^5^

## DISCUSSION

With growing national and international concerns, the Natural Calamity Act (1977) was revised to Disaster Risk Reduction and Management Act 2015 to address all four phases of the disaster cycle.^6,7^ Moreover, article number 35 in the constitution of Nepal 2015 states that every citizen shall have the right to free basic health services from the state and no one shall be deprived of emergency services. It also states that every citizen shall have equal access to health services.^6^ This holds even during the time of disaster. So, the country must be prepared to manage the fundamental health rights even during a period of crisis. This requires preparedness and a coordinated health effort.^8^ To facilitate this preparedness and coordination Health Emergency and Disaster Management Unit (HEDMU) was established. This unit is in the framework of the Ministry of Health and Population (MoHP) and is under the secretary for health. The unit coordinates through the health emergency operation center (HEOC). This center coordinates with the provincial health emergency operation center (PHEOC), other ministries, and other governmental and non-governmental agencies.^9^ At the central level, HEOC and at the province level PHEOC also coordinated with Hub and Satellite hospitals.

According to the disaster risk reduction and management act 2015, the highest body for disaster risk reduction, counteraction and recovery arethe National Council for Disaster Risk Reduction and Management (NCDRRM) which is under the Prime minister of Nepal.^10^ This council approves national policies and plans relating to disaster management. There is a Disaster Management Executive Committee (DMEC) under the home minister for the execution of policies and plans approved by the council. The Health Minister is a member of this committee. National disaster risk reduction authority (NDRRA) conducts activities for disaster risk reduction. Furthermore, there is a district disaster management committee under the chief district officer, the chief of health office is a member of this committee. At the local level, there is a local disaster management committee at the municipality level under the chairperson of Mayor.^10^ Operating centers which are the counterpart of health are National Emergency Operating Centre (NEOC), Provincial Emergency Operating Centre (PEOC), District Emergency Operating Centre (DEOC), and Local Emergency Operating Centre (LEOC). The NEOC coordinated with HEOC, National BhukampaMapan Kendra, TIA, REOC or DEOC, UN emergency center, Disaster Adhyananushadhan Kendra, NRCS, Nepal Army, APF, Nepal Police, Fire brigade, Meteorological division.

At the central level HEDMU coordinated with the Department of Health Province Disaster Management Committee (PDMC) in the Provincial Health Directorate and through HEOC with NEOC and Hub-satellite hospitals of the center. At the provincial level PDMC coordinates with health offices and hub and satellite hospitals. Coordination of Hub-satellite and health office is done through PHEOC. At the district level, district disaster management.

Implementation of the system and policy in the disaster is important so that the health care response system is prepared for disaster management. So, as per public health act 2018, chapter 6, section 48, number 2 the federal, provincial, and local level shall develop an emergency health plan and enforce it. Moreover, it is also stated in public health act chapter 2 section 4 number 1 that every health institution shall provide emergency health service as prescribed and this does not exclude during a disaster. Local government act section 3 (a) has also listed activities for disaster risk reduction and management at the local level.

The health cluster lead agency (CLA) serves as a bridge between the national and local health authorities and international and NGO humanitarian health actors. A key responsibility of CLA is to ensure that international humanitarian actors build on local capacities and that they develop and maintain appropriate links with relevant government and local authorities and local civil society organizations involved in health-related activities.

To strengthen the disaster management there is provision of developing trauma centers as per National health policy (section 6.3.2), national disease control center (section 6.4.1), emergency services at centers at all levels (Section 6.3.1), and one ambulance per local area (section 6.3.3.). Furthermore, during any disaster, basic health services (ten services) mandated by the government should not be withheld. So, a plan should be developed to run these ten basic health services smoothly. The right to safe motherhood and reproductive health Act, 2075 - chapter 2, Section 3, Number 3 secures the respective right of the female. So, a national strategy must address these issues. All hospitals must be prepared for disaster management plans as per “swasthya sewa sanchalan tatha gunasthar sambandhi byabastha ain” section 2 subsection 4b. So must have minimal standard as per MSS and there should be regular drill (section 2 subsection 7b).

In case of an infectious disease outbreak, there is a provision of the Infectious Disease Act 2020 (1964) which shall come into force immediately. According to this law, the Government of Nepal can take necessary actions to root out or prevent that disease and issue necessary orders applicable to the general public. It also states that the government may issue necessary orders for examining animals, birds being transported on foot or by any means of conveyance or of any passengers and can even hold them in quarantine in hospitals or other places.^11^

In case of a crisis that might occur in public security, interest, and community health, procurement can be made immediately as per Public Procurement Rules 2064 (2007) Chapter 15.^12^

## WAY FORWARD

Nepal has sufficient guiding documents to guide disaster management. Preparing health care facilities for disaster, planning effective coordination and resource sharing is important for preparedness. Moreover, in resource-limited countries like Nepal, it is a daily emergency.

## Conflict of Interest

**None.**
